# Modulation of physiological reflexes by pain: role of the locus coeruleus

**DOI:** 10.3389/fnint.2012.00094

**Published:** 2012-10-17

**Authors:** Elemer Szabadi

**Affiliations:** Psychopharmacology Section, Division of Psychiatry, University of NottinghamNottingham, UK

**Keywords:** pain, locus coeruleus, fear-conditioning, acoustic startle reflex, pupillary light reflex

## Abstract

The locus coeruleus (LC) is activated by noxious stimuli, and this activation leads to inhibition of perceived pain. As two physiological reflexes, the acoustic startle reflex and the pupillary light reflex, are sensitive to noxious stimuli, this review considers evidence that this sensitivity, at least to some extent, is mediated by the LC. The acoustic startle reflex, contraction of a large body of skeletal muscles in response to a sudden loud acoustic stimulus, can be enhanced by both directly (“sensitization”) and indirectly (“fear conditioning”) applied noxious stimuli. Fear-conditioning involves the association of a noxious (unconditioned) stimulus with a neutral (conditioned) stimulus (e.g., light), leading to the ability of the conditioned stimulus to evoke the “pain response”. The enhancement of the startle response by conditioned fear (“fear-potentiated startle”) involves the activation of the amygdala. The LC may also be involved in both sensitization and fear potentiation: pain signals activate the LC both directly and indirectly via the amygdala, which results in enhanced motoneurone activity, leading to an enhanced muscular response. Pupil diameter is under dual sympathetic/parasympathetic control, the sympathetic (noradrenergic) output dilating, and the parasympathetic (cholinergic) output constricting the pupil. The light reflex (constriction of the pupil in response to a light stimulus) operates via the parasympathetic output. The LC exerts a dual influence on pupillary control: it contributes to the sympathetic outflow and attenuates the parasympathetic output by inhibiting the Edinger-Westphal nucleus, the preganglionic cholinergic nucleus in the light reflex pathway. Noxious stimulation results in pupil dilation (“reflex dilation”), without any change in the light reflex response, consistent with sympathetic activation via the LC. Conditioned fear, on the other hand, results in the attenuation of the light reflex response (“fear-inhibited light reflex”), consistent with the inhibition of the parasympathetic light reflex via the LC. It is suggested that directly applied pain and fear-conditioning may affect different populations of autonomic neurones in the LC, directly applied pain activating sympathetic and fear-conditioning parasympathetic premotor neurones.

## Introduction

The locus coeruleus (LC) has been implicated in a number of physiological and psychological functions. The LC plays an important role in the promotion and maintenance of arousal (Robbins, 1984; Berridge, 2008; Carter et al., 2010; Berridge et al., 2012). It is a wakefulness-promoting nucleus situated in a strategic position in the center of the arousal/sleep network, collecting information from both wakefulness- and sleep-promoting nuclei in the network. Excitatory outputs from the LC project directly to the cerebral cortex and other wakefulness-promoting nuclei, whereas inhibitory outputs project to sleep-promoting nuclei (Samuels and Szabadi, 2008a,b). The LC is involved in autonomic regulation: it contributes to sympathetic outflow by an excitatory projection to preganglionic sympathetic neurones, and modulates parasympathetic activity via an inhibitory projection to parasympathetic preganglionic neurones (Samuels and Szabadi, 2008a,b). LC activity also influences endocrine functions via connections to the hypothalamic paraventricular nucleus (PVN) and tuberoinfundibular area (Samuels and Szabadi, 2008a), and the LC has been shown to be involved in stress responses associated with the activation of the hypothalamic-pituitary-adrenal axis (Plotsky et al., 1989; Valentino and Van Bockstaele, 2008; Ulrich-Lai and Herman, 2009; Hermans et al., 2011). The LC plays an important role in the maintenance of muscle tone via an excitatory projection to motoneurones in the brainstem and the spinal cord (Samuels and Szabadi, 2008a). Due to its extensive connections to the amygdala, limbic system, and cerebral cortex, the LC has been implicated in a number of cognitive functions (in particular, attention) and emotions (anxiety, mood) (Robbins, 1984; Berridge and Waterhouse, 2003; Aston-Jones and Cohen, 2005; Sara, 2009). The LC is also involved in the processing of pain signals and the modulation of pain sensation (see below).

It is known that two physiological reflexes, the acoustic startle reflex, a somatic reflex, and the pupillary light reflex, an autonomic reflex, are both modulated by pain. In this review, the possible involvement of the LC in the effect of pain on these reflexes will be considered. Most of the experimental studies quoted involve acute pain evoked by noxious stimulation; when these studies are referred to, the stimulus is specified either in the text or in brackets after the reference. When the pain condition was chronic (e.g., pathological or neuropathic pain), this is made clear in the text.

## Processing of pain by the LC

The nociceptive neurones of both the trigeminal sensory nuclei in the brainstem and the dorsal horn of the spinal cord project, via the trigemino-thalamic and spino-thalmic pathways to the somatosensory nucleus of the thalamus, a major pain-processing subcortical relay nucleus. Pain signals from the thalamus reach the somatosensory area of the cerebral cortex, the site of the highest level of pain processing, via the powerful thalamocortical pathway (Kiernan, 2005). Apart from the somatosensory thalamus, the LC is also an important subcortical relay nucleus in the pain-processing system, channeling pain information to the somatosensory cortex. Like the thalamus, the LC receives nociceptive inputs from both the trigeminal sensory nuclei and the dorsal horn of the spinal cord (Craig, 1992). Furthermore, the LC projects to higher pain-processing structures, such as the somatosensory thalamus, via the coeruleo-thalamic pathway (Peschanski and Besson, 1984; Westlund et al., 1991; Voisin et al., 2005), and also directly to the cerebral cortex, via the coeruleo-cortical pathway (Nieuwenhuys, 1985). Although the LC innervates all areas of the neocortex, one of its main projection targets is the somatosensory cortex (Levitt et al., 1984; Gaspar et al., 1989). There is physiological evidence of the processing of pain signals, evoked by the electrical stimulation of the tooth pulp, by the LC, as revealed by recording the activity of single neurones in the somatosensory thalamus (Voisin et al., 2005).

There is extensive evidence showing that noxious stimulation results in an increase in LC activity. Noxious stimuli evoke an increase in the electrical activity of LC neurones, shown by both extracellular [Kimura and Nakamura, 1985 (tail pinch, air puff); Elam et al., 1986 (noxious heat); Rasmussen et al., 1986 (pinch); Hirata and Aston-Jones, 1994 (foot shock)] and intracellular [Sugiyama et al., 2012 (pinch)] recording. Painful stimulation leads to an increase in the expression of Fos, the protein product of the activation of the intermediate early gene c-Fos, a marker of neuronal activity [Bullitt, 1990 (noxious heat and cold, pinch); Pezzone et al., 1993 (electric shock); Palkovits et al., 1995 (subcutaneous formalin injection); Voisin et al., 2005 (electric tooth pulp stimulation); Wang et al., 2009 (colon distension)]. Noxious stimuli evoke an increase in the release of noradrenaline from the LC [Singewald et al., 1999 (air puff, noise stress); Kaehler et al., 2000 (tail pinch); Sajedianfard et al., 2005 (subcutaneous formalin injection)]. A number of neurotransmitters and neuromodulators have been implicated in the modulation of the activation of the LC by noxious stimuli. There is direct evidence indicating the involvement of glutamate [Hayashida et al., 2010 (hind paw pressure)] and extracellular signal-regulated kinase (ERK) [Imbe et al., 2009 (subcutaneous formalin injection)]. Furthermore, the involvement of a number of other neurotransmitters/neuromodulators has been suggested on the basis of more indirect evidence, such as GABA (Pan et al., 2002), opiates (Pan et al., 2004), purines (Khakpay et al., 2010), and cannabinoids (Carvalho and Van Bockstaele, 2012).

## Modulation of pain by the LC

The LC projects to pain-sensitive neurones of the trigeminal sensory nuclei, via the coeruleo-trigeminal pathway (Senba et al., 1981; Tsuruoka et al., 2003b) and the dorsal horn of the spinal cord, via the coeruleo-spinal pathway (Guyenet, 1980; Fritschy and Grzanna, 1990; Liu et al., 2007), and also to the pain-processing neurones of the somatosensory thalamus (Peschanski and Besson, 1984; Westlund et al., 1991; Voisin et al., 2005). Via these modulatory pathways, the LC exerts an inhibitory influence on pain sensation [(Maeda et al., 2009) (hind paw inflammation)]; for reviews see Willis and Westlund (1997); Pertovaara and Almeida (2006); Ossipov et al. (2010). The LC can be activated by noxious stimulation (see above), which leads to the inhibition of the pain evoked by a noxious stimulus, such as hind paw inflammation (Tsuruoka et al., 2003a; Maeda et al., 2009). LC activation by direct electrical stimulation also evokes an anti-nociceptive effect [Margalit and Segal, 1979 (hot plate); West et al., 1993 (noxious heat: foot withdrawal response)]. Interestingly, it has been reported that the anaesthetic gas nitrous oxide activates the LC, and this effect has been implicated in the analgesic effect of the drug [Sawamura et al., 2000 (tail flick, hot plate)]. However, there are exceptions: the LC may facilitate, rather than attenuate, chronic neuropathic pain [Brightwell and Taylor, 2009 (nerve injury-induced hyperalgesia)]. Furthermore, the α_2_-adenoceptor agonist clonidine, a drug known to reduce LC activity (Aghajanian and VanderMaelen, 1982; Williams et al., 1985; Fernández-Pastor et al., 2005), has a paradoxical analgesic effect [Sawynok and Reid, 1986 (tail flick); Sierralta et al., 1996 (acetic acid writhing test); Wang et al., 1998 (hot plate); Yoshikawa et al., 2001 (pain evoked by propofol injection in humans); Hauck et al., 2006 (electric shock to fingertips in humans)]. However, as α_2_-adrenoceptors occur not only on LC neurones, but at many other sites in the pain-processing/pain-modulating pathways (Pan et al., 2008; Ossipov et al., 2010), clonidine's analgesic effect may not be mediated via the LC. Indeed, it has been reported that while clonidine decreases the activity of LC neurones in rats subjected to noxious stimulation by subcutaneously injected formalin, it activates dorsal horn neurones in the spinal cord, implicating this latter action in the analgesic effect of clonidine (Fukuda et al., 2006).

Conditioned pain modulation, also referred to as “diffuse noxious inhibitory controls,” involves the application of diffuse relatively mild painful stimuli which lead to the attenuation of the sensation of pain evoked by a localized strong noxious stimulus [Pertovaara and Almeida, 2006 (review); Lewis et al., 2012 (noxious cold and pressure, ischaemic arm test in humans)]. This mechanism has been implicated in the analgesic effect of acupuncture [Bing et al., 1990 (noxious heat)]. It has recently been reported that repeated injections of diluted bee venom, which lead to the attenuation of both acute pain evoked by a noxious thermal stimulus and chronic (neuropathic) pain evoked by the ligation of the sciatic nerve, activate the LC (Kang et al., 2012). Therefore the analgesic effect of conditioned pain modulation may involve the activation of the pain-inhibiting pathways arising from the LC. Indeed, duloxetine, a noradrenaline reuptake inhibitor which potentiates noradrenergic neurotransmission, enhances conditioned pain modulation [Yarnitsky et al., 2012 (noxious cold)], whereas dexmedetomidine, an α_2_-adrenoceptor agonist which reduces LC activity, inhibits it [Baba et al., 2012 (electric tooth pulp stimulation)]. Conditioned pain modulation is a form of sensory gating, and it may be analogous to “prepulse inhibition,” when a weak stimulus applied within a time window attenuates the effect of a strong stimulus (Perlstein et al., 2001). Indeed, it has been reported that pain sensation evoked by electric shocks is subject to modulation by “prepulses” (Blumenthal et al., 2001). It is an intriguing possibility that the analgesic effect of prepulses, like that of conditioned pain modulation, may be related to the activation of the LC.

## Acoustic startle reflex

**Figure 1:**
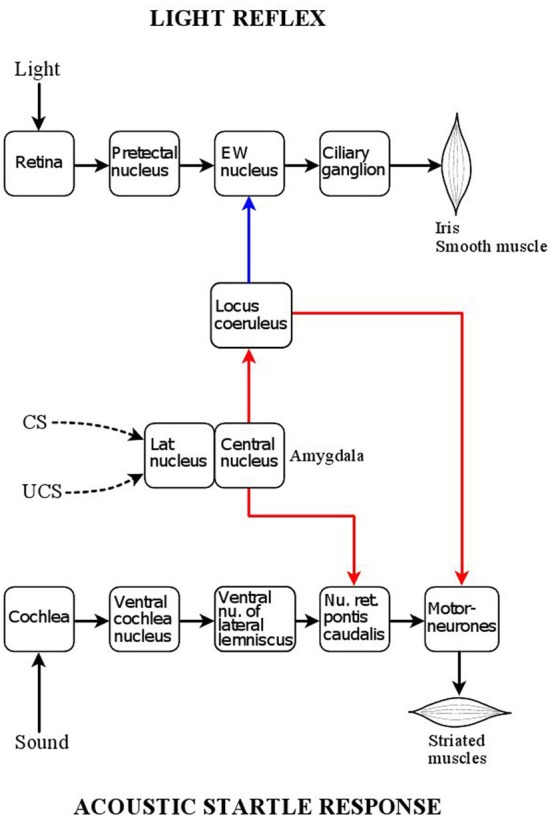
**Central position of the locus coeruleus in relation to the acoustic startle reflex and pupillary light reflex pathways.** Red: excitatory connections; blue: inhibitory connections. The *acoustic startle response* is triggered by a sound stimulus activating auditory receptors in the cochlea. Auditory signals are transmitted via two nuclei of auditory processing, the ventral cochlear nucleus and ventral nucleus of the lateral lemniscus, to a relay nucleus in the pontine reticular formation, nucleus reticularis pontis caudalis, which projects directly to bulbar and spinal motoneurones. The startle response consists of the sudden synchronized contraction of a large array of facial and skeletal muscles. The locus coeruleus has a facilitatory influence on the motor neurones via an excitatory noradrenergic output involving the stimulation of α_1_-adrenoceptors. Painful stimuli, via activation of the locus coeruleus can enhance the acoustic startle response (“sensitization”). The reflex response can also be enhanced by fear-conditioning via the amygdala. The lateral nucleus of the amygdala processes the association between aversive (painful) unconditioned (UCS) stimuli and neutral (e.g., light) conditioned (CS) stimuli, and the arising conditioned fear signal is transmitted, via the central nucleus of the amygdala, to the nucleus reticularis pontis caudalis, leading to the enhancement of the reflex response (“fear-potentiation”). The amygdala also projects to the locus coeruleus, whose activation by conditioned fear contributes to the fear-potentiation of the acoustic startle response. The *pupillary light reflex* is a parasympathetic autonomic reflex. Light signals stimulate photoreceptors in the retina which project, via melanopsin-containing intrinsically photosensitive retinal ganglion cells (ipRGCs), to the pretectal nucleus, a parasympathetic premotor nucleus: this leads to activation of the reflex pathway via the chain Edinger Westphal nucleus (preganglionic neurones) → ciliary ganglion (postganlionic neurones). The reflex response is the contraction of the smooth muscle fibres of the sphincter pupillae muscle, leading to pupil constriction (miosis). The locus coeruleus has inhibitory influence on the preganglionic neurones via a noradrenergic projection involving α_2_-adrenoceptors. As the locus coeruleus can be activated by the amygdala, it transmits conditioned fear signals to the Edinger Westphal nucleus, leading to the attenuation of the light reflex response by conditioned fear (“fear-inhibition”).

The acoustic startle reflex is the contraction of a large body of skeletal and facial muscles in response to a sudden loud auditory stimulus. The reflex pathway is simple, involving only four synapses (Figure 1). The reflex has been extensively studied in both experimental animals and humans (Yeomans and Frankland, 1996; Koch and Schnitzler, 1997; Koch, 1999). In human subjects the response recorded is usually the contraction of the orbicularis oculi muscle (eye-blink response) (Braff et al., 1978; Grillon et al., 1992; Kumari et al., 1996). The startle response is subject to sensory gating: a weak auditory stimulus applied within a time window prior to the index stimulus attenuates the response (“prepulse inhibition”) (Swerdlow et al., 1992; Perlstein et al., 2001; Samuels et al., 2007).

As the final neurone in the reflex pathway is a motoneurone, which is under noradrenergic influence (Funk et al., 2000; Heckman et al., 2009; Noga et al., 2011), the startle reflex is liable to be modulated by LC activity. Indeed, experimental lesioning of the LC has been reported to result in a reduction in the amplitude of the startle response (Adams and Geyer, 1981).

It is well documented that the LC sends excitatory projections, operating via the stimulation of α_1_-adrenoceptors, to motoneurones in both the brainstem and the spinal cord (Samuels and Szabadi, 2008a). The noradrenergic projection to motoneurones plays an important role in the maintenance of muscle tone: when LC activity is suspended, as during rapid eye movement sleep (Gottesmann, 2011) or attacks of cataplexy (Wu et al., 1999) total atonia ensues (Peever, 2011).

LC neurones are under auto-regulation via inhibitory somato-dendritic α_2_-adrenoceptors that dampen neuronal firing as activity increases (Huang et al., 2012). This mechanism may underlie the observation that the LC “switches off” when very high firing frequencies are attained in response to stimulation (Carter et al., 2010). Furthermore, it has been reported that narcolepsy is associated with an increase in the number of α_2_-adrenoceptors on LC neurones (Fruhstorfer et al., 1989): this could lead to increased auto-inhibition and the propensitiy of LC neurones to cease to fire when stimulated, as seen in attacks of cataplexy (Wu et al., 1999). Therefore, LC activity could be modified by experimental manipulation of central α_2_-adrenoceptors, which in turn could lead to changes in the acoustic startle response. Genetic manipulation of central α_2C_-adrenoceptors has been reported to be associated with changes in the acoustic startle response in mice: targeted inactivation of the gene encoding the receptor (α_2C_-KO) leading to enhancement, and over-expression of the receptor to attenuation of the startle response (Sallinen et al., 1998). α_2_-Adrenoceptor agonists, such as clonidine, that are known to inhibit LC activity (Aghajanian and VanderMaelen, 1982; Abercrombie and Jacobs, 1987; Fernández-Pastor et al., 2005), attenuate the acoustic startle response in both animals (Davis et al., 1979) and humans (Kumari et al., 1996; Abduljawad et al., 1997, 2001; Samuels et al., 2007). On the other hand, the α_2_-adenoceptor antagonist yohimbine, a drug that increases LC activity (Ivanov and Aston-Jones, 1995; Crespi, 2009), has been reported to facilitate the acoustic startle response in humans (Morgan et al., 1993). While drugs targeted at α_2_-adrenoceptors are likely to have a direct effect on LC activity, a number of drugs may modify LC activity indirectly, by modulating excitatory and inhibitory inputs to the LC. Thus the wakefulness-promoting drug modafinil facilitates the acoustic startle response in humans (Samuels et al., 2007), probably by potentiating the dopaminergic excitation of LC neurones (Hou et al., 2005). Indeed, the activation of the LC by modafinil has been demonstrated by fMRI in human subjects (Minzenberg et al., 2008). On the other hand, diazepam has been shown to reduce the amplitude of the acoustic startle response in both animals (Berg and Davis, 1984) and humans (Abduljawad et al., 1997, 2001). This effect of diazepam has been attributed to a reduction in LC activity associated with sedation (Samuels et al., 2007; Samuels and Szabadi, 2008b). However, it should be noted that the effect of diazepam on LC activity is likely to be indirect, since GABA receptors in the LC have been reported to be insensitive to diazepam (see section “Effect of Pain on Pupil Diameter: Reflex Dilation,” below). As the level of arousal is closely associated with LC activity (Samuels and Szabadi, 2008b), drugs known to facilitate the acoustic startle response, in general, are stimulants, whereas drugs inhibiting it are sedatives (Samuels et al., 2007).

The acoustic startle response has an autonomic component: the auditory stimulus also evokes a sympathetic response, including increases in blood pressure and heart rate (Baudrie et al., 1997; Holand et al., 1999; Eder et al., 2009) and sweat gland activity (Samuels et al., 2007). Interestingly, the autonomic component of the startle reflex, like the motor component (see above), is subject to prepulse modulation (Samuels et al., 2007; Eder et al., 2009). Although the exact connections of this “sensorysympathetic reflex” are not known, it is likely that a number of premotor sympathetic nuclei, including the ventrolateral medulla (Holand et al., 1999), the LC and the hypothalamic paraventricluar nucleus (Samuels et al., 2007) are involved.

### Effect of pain: sensitization

It has been reported that *acutely applied noxious stimuli*, such as foot shocks in rats (Davis, 1989; Fendt et al., 1994a,b; Krase et al., 1994) and noxious heat in humans (Grombez et al., 1997), increase the amplitude of the acoustic startle response (“sensitization”) (for reviews, see Koch and Schnitzler, 1997; Fendt and Fanselow, 1999; Koch, 1999). Interestingly, the presence of *chronic pain*, such as functional abdominal pain in children, can also lead to sensitization of the acoustic startle response (Bakker et al., 2010). The degree of sensitization seems to be related to the intensity of the noxious stimulus, such as electric shock: stronger stimuli evoke more severe pain accompanied by larger increases in the amplitude of the startle response (Duker et al., 2004).

The phenomenon of sensitization is consistent with the enhancement of LC activity evoked by the noxious stimulus, which in turn would lead to increased motoneurone response at the final step in the acoustic startle response pathway (see above, and Figure 1). This mechanism may provide the physiological basis for the proposal of Davis (1989) that sensitization was a simple “unlearned” or “unconditioned” response. However, this view has been challenged (Richardson, 2000): since pain evoked by a noxious stimulus is fear-inducing, sensitization may not be fundamentally different from fear-potentiation (see, section “Effect of Conditioned Fear: Fear-Potentiation,” below). Indeed, there is evidence that the effect of pain on the acoustic startle response may be susceptible to contextual factors, suggesting a conditioning mechanism (Richardson and Elsayed, 1998). This may explain, that in some situations sensitization could not be observed [Horn et al., 2012a,b (noxious heat in humans)], or that the noxious stimulus inhibited, rather than potentiated, the startle response [Sorenson and Swerdlow, 1982 (tail pinch in rats); Tavernor et al., 2000 (noxious cold in humans)].

The overlap between the mechanisms underlying sensitization and fear-potentiation is further strengthened by the observation that the amygdala, a structure essential for fear-conditioning (see below, and Figure 1), is also involved in sensitization (Hitchcock et al., 1989; Fendt et al., 1994b; Krase et al., 1994). The involvement of the LC in sensitization is rather complex. Pain may activate the LC both directly (see above) and indirectly via the amygdala, which in turn projects to the LC (Figure 1) (Samuels and Szabadi, 2008a; Reyes et al., 2011); LC activation would lead to the potentiation of motoneurone activity, resulting in the enhancement of the startle response. There is also a reciprocal connection between the LC and the amygdala: the LC does not only receive an input from the amygdala, but it also projects to it (Samuels and Szabadi, 2008a). The reciprocal connection between the LC and the amygdala provides the basis for a positive feed-back mechanism: by projecting to the amygdala, the LC can enhance its own activation by this structure. Indeed, it has been shown that noradrenaline release in the amygdala is involved in the sensitization of the acoustic startle response (Fendt et al., 1994b). Finally, LC activation by pain may increase the sensitivity of the startle reflex to auditory stimuli via an excitatory LC projection to cochlear nuclei (Kromer and Moore, 1976; Ebert, 1996; Gómez-Nieto et al., 2008), resulting in enhancement of the reflex response.

### Effect of conditioned fear: fear-potentiation

Fear-conditioning is based on associative learning (“Pavlovian conditioning”): pairing of a noxious (“unconditioned”) stimulus (US) with a neutral (“conditioned”) stimulus (CS), results in the development of the ability of the CS to evoke the response to the US (see e.g., Fendt and Fanselow, 1999). There is a large body of evidence that the acoustic startle response is subject to modulation not only by pain (see section “Modulation of Pain by the LC”, above), but also by conditioned fear arising from the prior pairing of a painful stimulus with a neutral stimulus (e.g., light). Conditioned fear leads to the enhancement of the acoustic startle response (“fear-potentiation”) (Davis, 1992; Davis et al., 1993; Koch and Schnitzler, 1997; Fendt and Fanselow, 1999; Koch, 1999). Fear-potentiation of the acoustic startle reflex has been studied extensively in both animals (Davis et al., 1993; Koch, 1999) and humans (Grillon et al., 1991; Bitsios et al., 1999a,b; Scaife et al., 2005; Hubbard et al., 2011). The unconditioned stimulus used in these experiments is usually electric shock: foot shock in animals and shock to the volar surface of the wrist in humans. It has been shown that the brain structure processing the association between US and CS is the lateral nucleus of the amygdala, which is connected to the central nucleus of the amygdala, the structure projecting to the caudal pontine reticular nucleus, a major relay nucleus in the acoustic startle reflex pathway (Figure 1) (Davis, 1992; Davis et al., 1993). The activity of neurones in the amygdala is susceptible to modulation by conditioned fear, as shown by increases in their firing rate [Pascoe and Kapp, 1985 (electric shock)] and in the expression of Fos in this structure (Davis et al., 1993). As discussed above (see “Modulation of Pain by the LC”), the central nucleus of the amygdala also projects to the LC, which in turn influences the activity of the motor neurone pool, the final step in the reflex pathway. There is evidence that the activity of the LC, like that of the amygdala, is subject to modulation by fear conditioning: neutral stimuli, previously associated with painful ones (i.e., electric shocks), increase LC activity, as shown by increases both in neuronal firing rate (Rasmussen and Jacobs, 1986) and Fos expression (Pezzone et al., 1993; Ishida et al., 2002; Liu et al., 2003).

A number of drugs can modify the fear-potentiated startle response (Davis, 1992; Davis et al., 1993; Fendt and Fanselow, 1999). It is likely that different drugs may act at specific sites within the reflex pathway. It is well documented that benzodiazepines attenuate or even block the response, both in animals (Davis, 1979; Berg and Davis, 1984; Hijzen and Slangen, 1989; Davis et al., 1993) and humans (Patrick et al., 1996; Bitsios et al., 1999a; Graham et al., 2005; Scaife et al., 2005). Although these drugs may act at a number of sites in the brain, it is likely that an action at the level of the amygdala is relevant. It has been reported that diazepam can selectively block the acquisition but not the expression of fear potentiation (Scaife et al., 2005, 2007), suggesting interference with the processing of the association between US and CS, which is known to be localised in the amygdala (Davis, 1992; Davis et al., 1993). The α_2_-adrenoceptor agonist clonidine is also a potent inhibitor of the fear-potentiated startle response (Davis et al., 1979). As a major action of clonidine is the inhibition of LC activity (Aghajanian and VanderMaelen, 1982; Abercrombie and Jacobs, 1987; Fernández-Pastor et al., 2005), the inhibition of the fear-potentiated startle by this drug corroborates the evidence about the involvement of the LC in fear-potentiation. Interestingly, the α_2_-adrenoceptor antagonist yohimbine, a drug known to stimulate the LC (Ivanov and Aston-Jones, 1995, Crespi, 2009), has the opposite effect: it facilitates fear-potentiation (Davis et al., 1979).

## Pupillary light reflex

**Figure 2:**
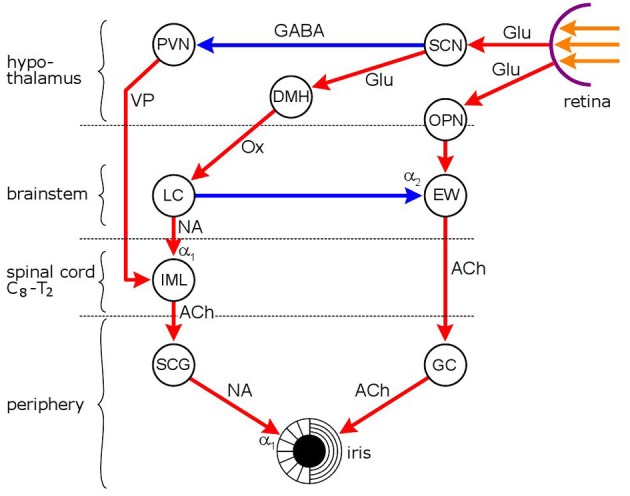
**Neuronal network mediating the effect of light on pupil diameter.** Red: excitatory connections, blue: inhibitory connections. Hypothalamic nuclei: SCN, suprachiasmatic nucleus; PVN, paraventricular nucleus; DMH, dorsomedial hypothalamus; autonomic premotor nuclei: OPN, olivary pretectal nucleus; LC, locus coeruleus; parasympathetic nucleus/ganglion: EW, Edinger Westphal nucleus; GC, ganglion ciliare; sympathetic nucleus/ganglion: IML, intermedio-lateral column of spinal cord; SCG, superior cervical ganglion. Neurotransmitters: Glu, glutamate; GABA, γ-amino-butyric acid; VP, vasopressin; Ox, orexin; ACh, acetylcholine; NA, noradrenaline. Adrenoceptors: α_1_, excitatory and α_2_, inhibitory. Pupil diameter reflects the relationship between two opposing smooth muscles, the dilator pupillae, innervated by the sympathetic, and sphincter (constrictor) pupillae (innervated by the parasympathetic). The locus coeruleus functions as both a sympathetic and a parasympathetic premotor nucleus: it stimulates preganglionic sympathetic neurones in the IML and inhibits preganglionic parasympathetic neurones in the EW. The pupillary light reflex is a parasympathetic reflex: light signals from the retina stimulate the chain OPN → EW → GC, leading to pupil constriction. Light also has an indirect effect on sympathetic activity via the SCN: sympathetic activity is inhibited via an inhibitory output to the PVN. Light-evoked sympatho-inhibition, however, is likely to be attenuated by sympatho-excitation mediatied via the SCN → DMH → LC route.

**Figure 3:**
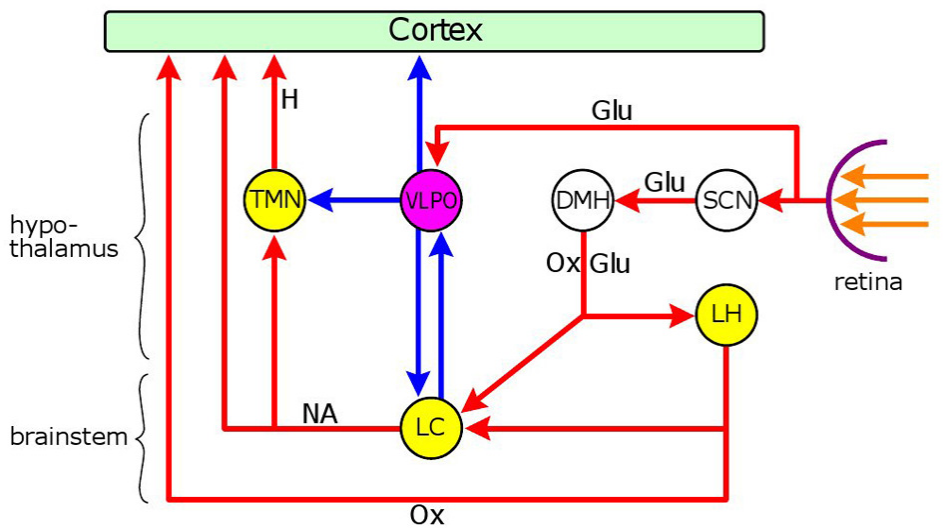
**Neuronal network mediating the dual effect of light on arousal.** Nuclei: yellow: wakefulness-promoting, purple: sleep-promoting. Connections: red: excitatory, blue: inhibitory. Hypothalamic nuclei: SCN, suprachiasmatic nucleus; DMH, dorso-medial hypothalamus; LH, lateral hypothalamic area; VLPO, ventrolateral preoptic nucleus; TMN, tuberomamillary nucleus; brainstem nucleus: LC, locus coeruleus. Neurotransmiters: Glu, glutamate; Ox, orexin; NA, noradenaline; H, histamine. Light reaching the retina has a sleep-promoting effect via the excitatory output of melanopsin-containing intrinsically photosensitive retinal ganglion cells (ipRGCs) to the VLPO, the major sleep-promoting nucleus. GABAergic inhibitory neurones in the VLPO project to the cerebral cortex, and two major wakefulness-promoting nuclei, the TMN and LC. Light also evokes a wakefulness-promoting effect via the SCN which can stimulate orexinergic and glutamatergic neurones in the DMH that project to two wakefulness-promoting nuclei, the LC and LH. There is a reciprocal inhibitory connection between the LC and the VLPO: GABAergic neurones in the VLPO inhibit the LC, and noradrenergic neurones in the LC inhibit the VLPO via the stimulation of α_2_-adrenoceptors. The LC also stimulates the cortex and the TMN via excitatory outputs involving α_1_-adrenoceptors. The orexinergic neurones of the LH send excitatory outputs to the LC and the cerebral cortex. The overall effect of light on arousal depends on the relationship between the two light-sensitive arousal systems. In nocturnal animals light is sleep-promoting due to the predominant effect of the activation of the VLPO by light. In diurnal animals, on the other hand, the sleep-promoting effect of VLPO activation by light, is likely to be superseded by the wakefulness-promoting influence of the activation of the LC and LH via the SCN → DMH → LC/LH route.

The pupil is an aperture in a diaphragm, the iris, whose function is to control the amount of light reaching the retina. The iris contains two smooth muscles, the sphincter (or constrictor) pupillae, and the dilator pupillae. The two muscles receive opposing parasympathetic and sympathetic innervations, and thus pupil diameter at any time reflects the intricate balance between the two output systems (Samuels and Szabadi, 2008a,b). The pupillary light reflex, an autonomic reflex, consists of the constriction of the pupil in response to a light stimulus. The reflex pathway is shown in Figure 2. Three photoreceptors are involved: rods and cones that project to a subgroup of retinal ganglion cells (“intrinsically photosensitive ganglion cells, ipRGCs”) containing the photopigment melanopsin. The ipRGCs constitute the third photoreceptor (Kawasaki and Kardon, 2007). The ipRGCs mediate not only the pupillary light reflex, but also all other “non-image-forming” (NIF) visual functions, such as the regulation of circadian rhythms, sleep/arousal and autonomic/endocrine activity. The ipRGCs give rise to two pathways: one to the olivary pretectal nucleus (OPN), a preomotor nucleus in the parasympathetic light reflex pathway, and another one (retino-hypothalamic tract) to two hypothalamic nuclei, the suprachiasmatic nucleus (SCN) and the ventrolateral preoptic nucleus (VLPO) (Figures 2 and 3) (Lu et al., 1999; Gooley et al., 2003; Güler et al., 2008). The SCN is responsible for the generation of circadian rhythms and modulation of autonomic and endocrine functions (Kalsbeek et al., 2000, 2006; van Esseveldt et al., 2000), whereas the VLPO is a major sleep-promoting nucleus (Lu et al., 1999; Samuels and Szabadi, 2008b).

The pupillary light reflex is a parasympathetic reflex: the OPN projects to the preganglionic parasympathetic cholinergic neurones located in the Edinger-Westphal nucleus (EWN) of the midbrain; the EWN innervates the postganglionic cholinergic neurones in the ciliary ganglion; the postganglionic neurones innervate the constrictor pupillae muscle.

The sympathetic innervation of the iris does not directly participate in the light reflex: its main function is the adjustment of pupil diameter to the level of background illumination. The sympathetic preganglionic neurones are located in the intermedio-lateral column of the cervico-thoracic spinal cord, and project to the noradrenergic postganglionic neurones in the superior cervical ganglion, which innervate the dilator pupillae muscle. A number of premotor nuclei modulate the activity of the preganglionic sympathetic neurones, of which the PVN of the hypothalamus and the LC are the most important (Figure 2) (Samuels and Szabadi, 2008a).

### Modulation of the pupillary light reflex by the LC

The LC plays a dual role in pupillary control: it contributes to sympathetic outflow via an excitatory projection to preganglionic sympathetic neurones, and it also modulates the parasympathetic output to the iris via an inhibitory connection to the EWN. The close relationship between LC activity and pupillary function is illustrated by the observation that fluctuations in the firing rate of LC neurones are closely paralleled by fluctuations in the diameter of the pupil (Aston-Jones and Cohen, 2005).

Via its projection to the EWN, the LC inhibits the pupillary light reflex. It has been shown that anxiety, an emotional state known to be associated with LC activation (Millan, 2003), leads to attenuation of the pupillary light reflex response (Bakes et al., 1990). Similarly, drugs that are likely to increase noradrenergic output to the EWN (e.g., noradrenaline re-uptake inhibitors), decrease the amplitude of the light reflex response (Theofilopoulos et al., 1995; Bitsios et al., 1999b; Szabadi and Bradshaw, 2000). On the other hand, the α_2_-adrenoceptor agonist clonidine, a drug “switching off” LC activity by stimulating inhibitory autoreceptors on LC neurones (Aghajanian and VanderMaelen, 1982; Abercrombie and Jacobs, 1987; Fernández-Pastor et al., 2005), causes pupil constriction (miosis), due partly to the inhibition of sympathetic outflow and partly to the removal of the noradrenergic inhibition of the EWN (Samuels and Szabadi, 2008b). However, it should be noted that the effect of clonidine depends on the species studied: while in diurnal animals (man, dog, and rabbit) clonidine causes miosis, in nocturnal animals (cat, rat, and mouse) it causes pupil dilation (mydriasis). This latter effect is likely to reflect the activation of postsynaptic inhibitory α_2_-adrenoceptors on EWN neurones in preference to the stimulation of presynaptic autoreceptors on LC neurones (Samuels and Szabadi, 2008b).

### Modulation of LC activity by light

Interestingly, light does not only evoke the light reflex, but also influences the sympathetic output to the iris. Light, by inhibiting the PVN, a major sympathetic premotor nucleus, via the SCN, exerts an inhibitory effect on the activity of preganglionic sympathetic neurones in the IML of the upper thoracic (T1-T3) spinal cord, synapsing in the superior cervical ganglion (Kalsbeek et al., 2000; Perreau-Lenz et al., 2003): this effect leads to pupil constriction (Passatore, 1976; Passatore and Pettorossi, 1976; Szabadi et al., 2010) and inhibition of melatonin synthesis (Nishino et al., 1976; Kalsbeek et al., 1999; Zeitzer et al., 2000). However, light may also have a stimulatory effect on sympathetic activity, by stimulating the LC via the SCN and the dorso-medial hypothalamus (DMH) (Figure 3) (Aston-Jones et al., 2001; Aston-Jones, 2005; Gonzalez and Aston-Jones, 2006). Indeed, it has been shown by fMRI in humans that short wavelength (blue) light, that preferentially stimulates the melanopsin-containing photoreceptors of ipRGCs (Kawasaki and Kardon, 2007), causes activation in a brain stem area corresponding to the LC (Vandewalle et al., 2007). Interestingly, the overall effect of light on sympathetic activity is inhibitory at the upper thoracic (T1–T3) levels of the spinal cord, whereas it is stimulatory at the levels of lower thoracic segments, leading to stimulation of cardiovascular activity (Michimori et al., 1997; Scheer et al., 1999; Cajochen et al., 2005), and corticosterone secretion from the adrenals (Niijima et al., 1992; Ishida et al., 2005; Hatanaka et al., 2008). The effect of light on cardiovascular activity seems to be species-dependent: while it is stimulatory in diurnal animals, it is inhibitory in nocturnal animals (Scheer et al., 2001), consistent with the close association of cardiovascular activity with the level of arousal (see below). The spinal-segment-related effect of light on sympathetic outflow, which conforms to the principle of sympathetic organization according to “tissue-specific sympathetic output pathways” (Morrison, 2001), has been attributed to the operation of region-specific specialization in the SCN (Scheer et al., 2003).

The modulation of LC activity by light is likely to be different between nocturnal and diurnal animals, as highlighted by the effect of light on arousal: in nocturnal animals, light is sleep-promoting (Altimus et al., 2008; Tsai et al., 2009), whereas in diurnal animals, including man, is wakefulness-promoting (Cajochen et al., 2005; Lockley et al., 2006; Revell et al., 2006). In both nocturnal and diurnal animals light has a dual effect: promoting sleep via the activation of the VLPO, and wakefulness via the activation of the SCN-MDH-LC circuit (Figure 3), the overall effect depending on the relationship between the two opposite influences. Therefore in diurnal animals the wakefulness-promoting effect of the activation of the SCN-MDH-LC circuit is likely to supersede the sleep-promoting effect arising from the activation of the VLPO. It is an intriguing possibility that the greater sensitivity of presynaptic α_2_-adrenoceptors on LC neurones to clonidine, compared to postsynaptic receptors, in diurnal animals (see section “Modulation of the Pupillary Light Reflex by the LC,” above), may be related to the more pronounced activation of the LC by light in these species.

The activation of the LC by light may have implications for the processing and modulation of pain signals. It has been reported that diurnal animals, including man, are less sensitive to pain during day time (Rigas et al., 1990; Göbel and Cordes, 2005) whereas the opposite seems to be the case in nocturnal animals (Kavaliers and Hirst, 1983; Kavaliers et al., 1984; Yoshida et al., 2003). The time-of-day-related variations in pain sensitivity have been attributed to circadian variations in endorphin synthesis (Kavaliers and Hirst, 1983; Rasmussen and Farr, 2003). However, they may also reflect diurnal variations in LC activity: in diurnal animals the LC is maximally active during day time, and LC activity is known to be associated with an analgesic effect (see section “Processing of Pain by the LC,” above). Furthermore, it has been reported that exposure to bright sunlight reduces post-operative pain in surgical patients (Walch et al., 2005), consistent with enhanced LC activity.

### Effect of pain on pupil diameter: reflex dilation

“Any sensory stimulus (with the exception of light) can elicit pupillary dilation” (Loewenfeld, 1993), and noxious stimuli are no exception. It is well documented that painful stimuli dilate the pupil in both animals and humans. In animals, the noxious stimulus used was electric stimulation of the sciatic nerve (Koss et al., 1984; Hey et al., 1985; Koss, 1986; Hey and Koss, 1988; Yu and Koss, 2003, 2004), and in humans either noxious cold (“cold pressor test”) (Tassorelli et al., 1995; Tavernor et al., 2000; Hou et al., 2007) or electric shock (Chapman et al., 1999; Larson and Talke, 2001; Yang et al., 2003; Walter et al., 2005; Oka et al., 2007). As noxious stimulation activates the LC (see section “Processing of Pain by the LC,” above), and the LC is intimately involved in pupillary control (see section “Modulation of the Pupillary Light Reflex by the LC” and Figure 2), it is likely that reflex dilation of the pupil (mydriasis) evoked by painful stimuli is mediated by the LC. Indeed, it has been reported that reflex pupil dilation to noxious stimuli is abolished following central noradrenaline depletion by reserpine or alpha-methyl-para-tyrosine (Koss et al., 1984). An increase in LC activity can lead to pupil dilation in two ways: by increasing sympathetic outflow to the iris, via an excitatory output to sympathetic preganglionic neurones in the spinal cord, and by inhibiting parasympathetic output, via an inhibitory connection to parasympathetic preganglionic neurones in the EWN.

There is direct evidence that painful stimulation increases impulse flow in sympathetic fibres innervating the iris [Passatore, 1976 (pinch, corneal touch)]. The importance of sympathetic activation in reflex pupil dilation to painful stimuli has been demonstrated in humans. Firstly, the cold pressor test (plunging one hand into ice cold water), which constitutes an intense painful stimulus (Yarnitsky and Ochoa, 1990) and is a powerful sympathetic activator (Seals, 1990), evokes pupil dilation (Tassorelli et al., 1995; Tavernor et al., 2000; Hou et al., 2007). Secondly, reflex dilation of the pupil can be antagonized by topical application of α_1_-adrenoceptor antagonists (e.g., thymoxamine: Tassorelli et al., 1995; dapiprazole: Yang et al., 2003; Hou et al., 2007) to the cornea: these drugs would block the effect of noradrenaline released by sympathetic stimulation. Thirdly, the α_2_-adrenoceptor agonist dexmedetomidine has been reported to reduce reflex dilation of the pupil (Larson and Talke, 2001), consistent with a central sympatholytic effect resulting from the inhibition of the LC by dexmedetomidine (Chiu et al., 1995).

As benzodiazepines have been reported to inhibit sympathetic activity (Marty et al., 1986; Ikeda et al., 1994; Kitajima et al., 2004), it would be expected that these drugs, like α_2_-adrenoceptor agonists, would antagonize reflex dilation of the pupil. However, in a study examining the effect of diazepam on pupillary and cardiovascular functions, diazepam failed to attenuate reflex dilation of the pupil, while it antagonized the increase in systolic blood pressure, evoked by the cold pressor test (Hou et al., 2007). Interestingly, a similar dissociation between the effects of diazepam on pupil dilation and increase in blood pressure evoked by central sympathetic stimulation has been reported in cats: pupil dilation remained unaffected while the blood pressure rise was antagonized (Sigg and Sigg, 1969). These observations suggest that different sections of the sympathetic nervous system, one diazepam-insensitive and another one diazepam-sensitive, may be responsible for mediating pupillary and cardiovascular responses (Sigg et al., 1971). The diazepam-insensitive system, responsible for pupillary control, is likely to involve the LC, since LC neurones, although they contain GABA_A_ receptors (Kaur et al., 1997; Chen et al., 1999), have been reported to be insensitive to diazepam (Chen et al., 1999). On the other hand, a likely candidate for the diazepam-sensitive system is the one originating in the PVN. The role of the PVN in cardiovascular control is well established (Coote, 2005; Li and Pan, 2007; Womack et al., 2007; Nunn et al., 2011), and it has been shown that sympathetic premotor neurones in the PVN are inhibited by diazepam (Zahner et al., 2007).

While noxious stimulation in humans results in the activation of the sympathetic outflow to the iris, there is no evidence of any alteration in the parasympathetic output to the iris. Indeed, it has been reported that the pupillary light reflex response, a sensitive index of LC activity (see section “Effect of Pain: Sensitization,” above), remains unaffected by noxious stimulation (Tavernor et al., 2000; Hou et al., 2007). Therefore, pupillary reflex dilation in humans seems to be a pure sympathetic response. Interestingly, the same pattern seems to apply to rabbits, in whom no parasympathetic contribution to pupillary reflex dilation could be demonstrated, and thus the response appears to be mediated entirely by the sympathetic (Yu and Koss, 2003, 2004).

On the other hand, a different pattern has been reported in cats and rats (Koss et al., 1984; Hey et al., 1985; Koss, 1986; Hey and Koss, 1988). In these species, pupil dilation evoked by noxious stimulation is preserved after cutting the sympathetic nerve innervating the dilator muscle of the iris; the α_2_-adrenoceptor agonist clonidine, like noxious stimulation, dilates the pupil; the α_2_-antagonist yohimbine antagonizes the pupil dilatory effects of both clonidine and noxious stimulation. On the basis of these observations, Koss and his colleagues concluded that reflex dilation of the pupil was mediated by noradrenergic inhibition of the preganglionic parasympathetic neurones in the EWN, with little contribution by the sympathetic (Koss, 1986).

The apparent species difference in the mediation of reflex dilation of the pupil evoked by noxious stimuli is consistent with the suggestion that separate populations of LC neurones may function as sympathetic and parasympathetic premotor neurones (Samuels and Szabadi, 2008a). Thus in diurnal animals (rabbit, man) painful stimuli my activate predominantly the sympathetic premotor neurones in the LC, whereas in nocturnal animals (rat, cat) they may activate predominantly the parasympathetic premotor neurones. This species difference in the effect of painful stimuli on the LC is paralleled by species differences in the effect of light on arousal (wakefulness-promoting in diurnal animals, sleep-promoting in nocturnal animals) and cardiovascular activity (stimulatory in diurnal animals, inhibitory in nocturnal animals), and is also reflected in the site of action of α_2_-adrenoceptor agonists on central noradrenergic neurones (predominantly presynaptic in diurnal animals, predominantly postsynaptic in nocturnal animals) (see sections “Modulation of the Pupillary Light Reflex by the LC” and “Modulation of LC Activity by Light,” above).

### Effect of conditioned fear on light reflex: fear inhibition

As conditioned fear potentiates the acoustic startle reflex, and the activation of the LC is likely to contribute to this effect (see section “Effect of Conditioned Fear: Fear-Potentiation,” above), it could be predicted that conditioned fear may also affect the pupillary light reflex which is under inhibitory noradrenergic control from the LC (see section “Modulation of the Pupillary Light Reflex by the LC,” above). This prediction seemed to be supported by the observation that patients suffering from general anxiety disorder had attenuated light reflex responses, with relatively little change in resting pupil diameter, compared to healthy controls (Bakes et al., 1990). The reduction in the amplitude of the light reflex response in the anxious patients could be interpreted as the consequence of enhanced LC activity associated with anxiety (Millan, 2003).

Using a protocol identical to that which had been applied in a study of the effect of conditioned fear on the acoustic startle reflex in human volunteers, using electric shock as the conditioned stimulus (Grillon et al., 1991), it was shown that conditioned fear caused an increase in pupil diameter and a reduction in the amplitude of the light reflex response, associated with increases in subjective ratings of alertness and anxiety (Bitsios et al., 1996). In a subsequent experiment, the effect of conditioned fear on the two reflexes was compared within the same subjects and sessions. Conditioned fear had opposite effects on the two reflexes: the acoustic startle reflex was potentiated, while the pupillary light reflex was inhibited (Bitsios et al., 1999a). Figure 1 illustrates the position of the LC in modulating both the acoustic startle reflex and the pupillary light reflex. In the case of both reflexes, the amygdala, the structure processing the association between US (e.g., pain) and CS (e.g., light) plays a central role: activation of the amygdala leads to the activation of the LC. LC activation, however, has opposite effects on the two reflexes: while it has a facilitatory effect on the acoustic startle reflex by enhancing the noradrenergic excitation of motoneurones via the stimulation of α_1_-adrenoceptors, it has an inhibitory effect on the pupillary light reflex by enhancing the noradrenergic inhibition of preganglionic parasympathetic neurones in the EWN via the stimulation of α_2_-adrenoceptors.

Like in the case of the fear-potentiation of the acoustic startle reflex (Grillon et al., 1991; Davis et al., 1993), anxiety plays an important role in the inhibition of the pupillary light reflex by conditioned fear. The anticipation of an aversive stimulus (i.e., pain-induced by an electric shock), leading to the inhibition of the light reflex, has been reported to be associated with increases in subjective ratings of anxiety (Bitsios et al., 1996, 1999a,b, 2004). Furthermore, the size of the effect of conditioned fear on the pupillary light reflex has been reported to be related to the pre-existing level of anxiety, subjects with higher levels of “state” anxiety showing larger effects (Bitsios et al., 2002).

Drugs that are known to have effects on the fear-potentiated acoustic startle reflex (see section “Effect of Conditioned Fear: Fear-Potentiation”, above), also modify the fear-inhibited light reflex. It has been reported that both clonidine (Bitsios et al., 1998a) and diazepam (Bitsios et al., 1998b, 1999a) can antagonize the effect of conditioned fear on the pupillary light reflex. As discussed in relation to the fear-potentiated startle reflex, clonidine may act directly on the LC, whereas the most likely site of action of diazepam is the amygdala.

Conditioned fear has a dual effect on the pupil: it causes a small increase in pupil diameter together with the inhibition of the light reflex response (Bitsios et al., 1996, 1999a,b, 2004). There are indications that the two effects may be mediated by separate mechanisms: the increase in pupil diameter can be evoked by the anticipation of a neutral (e.g., acoustic) stimulus, and is accompanied by an increase in the level of alertness, whereas the inhibition of the light reflex can be evoked by the anticipation of an aversive stimulus, and is accompanied by an increase in anxiety (Bitsios et al., 2004). These observations suggest that the effect of conditioned fear on pupil diameter may involve mainly the activation of sympathetic premotor neurones in the LC, that are also closely associated with the modulation of arousal. On the other hand, the effect of conditioned fear on the light reflex may mainly be due to the activation of parasympathetic premotor neurones in the LC, which may be closely associated with anxiety. This hypothesis is supported by the observation that the α_1_-adrenoceptor antagonist dapiprazole, which can block the effect of the sympathetic on the iris when applied topically to the cornea (Yang et al., 2003; Hou et al., 2007), has been reported to inhibit the effect of conditioned fear on pupil diameter without affecting its effect on the light reflex response (Giakoumaki et al., 2005).

## Conclusions and clinical implications

The two physiological reflexes, acoustic startle reflex and pupillary light reflex, considered in this review, are both sensitive to pain. The acoustic startle reflex, a somatic reflex, is enhanced by painful stimulation, whereas the pupillary light reflex, an autonomic reflex, is inhibited by it. Although the two reflexes have very different mechanisms and underlying neuronal circuitries, they are both under modulation by the LC, which itself is sensitive to noxious stimulation, and via its widespread projections influences many somatic (e.g., muscle tone) and autonomic (e.g., pupillary activity) functions.

Directly applied painful stimuli enhance the acoustic startle response (“sensitization”) by augmenting the facilitation of motoneurone activity by the LC. Noxious stimulation leads to pupil dilation (“reflex dilation”), which seems to be mediated by different mechanisms in diurnal and nocturnal animals. In diurnal animals, reflex dilation is likely to be due to the activation of sympathetic premotor neurones in the LC, whereas in nocturnal animals, it seems to be caused by the inhibition of parasympathetic premotor neurones in the LC. Directly applied pain has no effect on the pupillary light reflex in man (it has not been studied in animals).

Painful stimuli applied indirectly via fear-conditioning, when a neutral stimulus alone can evoke a state of anticipatory fear/anxiety following its prior association with an aversive (painful) stimulus, also enhance the acoustic startle reflex (“fear-potentiated startle”), but they inhibit the pupillary light reflex (“fear-inhibited light reflex”). Fear-conditioning is mediated by the amygdala which has an excitatory influence on the LC. LC activation by the amygdala enhances the noradrenergic facilitation of motoneurone activity, leading to augmentation of the potentiation of the acoustic startle reflex, arising from the stimulation of the pontine relay nucleus in the reflex pathway. LC activation by the amygdala leads to the attenuation of the pupillary light reflex due to enhancement of the noradrenergic inhibition of the preganglionic parasympathetic neurones in the light reflex pathway. Interestingly, there seems to be some selectivity in the action of pain applied directly or indirectly, via fear conditioning, on premotor autonomic neurones in the LC: while directly applied pain stimulates the sympathetic premotor neurones, leading to pupil dilation, in diurnal animals, fear-conditioning stimulates the parasympathetic premotor neurones, leading to attenuation of the light reflex response.

There are some clinical conditions, usually involving pain, fear/anxiety and/or stress, that may be associated with abnormalities of the two reflexes. Furthermore, it is likely that these abnormalities reflect altered LC activity. *Post-traumatic stress disorder (PTSD)* is “a chronic, debilitating psychiatric disorder that can follow exposure to extreme stressful experiences” (Stam, 2007). Cardinal features of the syndrome include hyperarousal, increased startle responses, re-experiencing the traumatic event (“flashbacks”), avoidance behavior (Marshall and Garakani, 2002; Stam, 2007). Apart from the reports by patients of an increased tendency to startle, there is also laboratory evidence of sensitization of the startle reflex in PTSD (Butler et al., 1990; Orr et al., 1995; Morgan et al., 1996). However, it should be noted that exaggerated startle responses have not been reported in every study in which PTSD patients were compared with healthy controls (Grillon et al., 1996; Siegelaar et al., 2006). In addition to the enhancement of the muscular startle response, as recorded by EMG, there is also evidence of increased autonomic startle reactivity, as measured by heart rate and skin conductance responses, in PTSD (Orr et al., 1995; Shalev et al., 2000; Orr et al., 2003; Elsesser et al., 2004; Siegelaar et al., 2006). Interestingly, the startle reactivity of PTSD patients has been shown to decline as symptoms subside in the course of therapy (Griffin et al., 2012). The augmented startle reactivity in PTSD patients may be related to increased LC activity. It is generally recognized that stress leads to the activation of the LC (Palkovits et al., 1995; Pacák and Palkovits, 2001; Valentino and Van Bockstaele, 2008). Furthermore, the clinical presentation of patients with PTSD is often complicated by pain and/or fear/anxiety, variables known to lead to the activation of the LC (see sections “Modulation of Pain by the LC,” “Effect of Pain: Sensitization,” and “Effect of conditioned fear: fear-potentiation,” above). Finally, there is evidence of enhanced noradrenergic activity in patients suffering from PTSD (Southwick et al., 1993; Jacobsen et al., 2001). *Chronic pain* has also been reported to be associated with exaggerated startle responses (Carleton et al., 2006; Bakker et al., 2010). Again, this observation may be related to enhanced LC activity, since pain is known to lead to the activation of the LC (see section “Modulation of Pain by the LC,” above). Furthermore, the indirect activation of the LC by pain, via the generation of fear and anxiety through the amygdala (see sections “Effect of Pain: Sensitization” and “Effect of conditioned fear: fear-potentiation,” above), may also play a role in the enhancement of the startle response in chronic pain conditions. Finally, *anxiety states* have been reported to lead to alterations of the two physiological reflexes. Both enhanced startle reflexes (Bakker et al., 2009; Reeb-Sutherland et al., 2009) and diminished pupillary light reflexes (Bakes et al., 1990) have been described in patients suffering from anxiety disorders. These observations are consistent with increased LC activity: the activation of the LC by anxiety is well documented (for reviews, see Millan, 2003; Samuels and Szabadi, 2008b).

In conclusion, the enhancement of the startle response in PTSD, chronic pain conditions and anxiety states, and the attenuation of the pupillary light reflex response in anxiety disorder, can be interpreted on the basis of the involvement of the LC in the neuronal circuits controlling these two reflexes.

### Conflict of interest statement

The author declares that the research was conducted in the absence of any commercial or financial relationships that could be construed as a potential conflict of interest.
